# Bufei Jianpi Granules Reduce Quadriceps Muscular Cell Apoptosis by Improving Mitochondrial Function in Rats with Chronic Obstructive Pulmonary Disease

**DOI:** 10.1155/2019/1216305

**Published:** 2019-01-06

**Authors:** Jing Mao, Ya Li, Suyun Li, Jiansheng Li, Yange Tian, Suxiang Feng, Xuefang Liu, Qingqing Bian, Junzi Li, Yuanyuan Hu, Lanxi Zhang, Huige Ji

**Affiliations:** ^1^College of Pharmacy, Henan University of Chinese Medicine, Zhengzhou 450046, China; ^2^Henan Key Laboratory of Chinese Medicine for Respiratory Disease, Henan University of Chinese Medicine, Zhengzhou, Henan 450046, China; ^3^Central Laboratory, Respiratory Pharmacological Laboratory of Chinese Medicine, The First Affiliated Hospital, Henan University of Chinese Medicine, 19 Renmin Road, Zhengzhou, Henan 450000, China; ^4^Respiratory Disease Institute, Department of Respiratory Disease, The First Affiliated Hospital, Henan University of Chinese Medicine, 19 Renmin Road, Zhengzhou, Henan 450000, China; ^5^Collaborative Innovation Center for Respiratory Disease Diagnosis and Treatment & Chinese Medicine Development of Henan Province, Henan University of Chinese Medicine, Zhengzhou, Henan 450046, China

## Abstract

**Background:**

Cell apoptosis is an important mechanism underlying skeletal muscle dysfunction in chronic obstructive pulmonary disease (COPD) patients, and mitochondrial dysfunction is recognized as a central aspect contributing to skeletal muscle deterioration. Bufei Jianpi granules have been confirmed effective for improving motor function in COPD patients, but the specific mechanism for this improved function remains unknown. This study explored the mechanisms by which Bufei Jianpi granules improve cell apoptosis and mitochondrial dysfunction in COPD.

**Methods:**

Sprague-Dawley rats were randomized into control, model, Bufei Jianpi, and aminophylline groups. A stable COPD rat model was induced with respective repeated cigarette smoke inhalation and intragastric bacterial infection, and rats were sacrificed after 20 weeks; the quadriceps muscle was harvested from each rat. Skeletal muscle mitochondria were extracted for measurements of mitochondrial membrane potential (MMP) and mitochondrial permeability transition pore openings (mPTPs). ATP levels were determined with a firefly luciferase-based ATP assay kit. The rates of cell apoptosis were determined by the transferase-mediated deoxyuridine triphosphate-biotin nick end labeling (TUNEL) method. Cyto C and caspase-3 mRNA and protein levels were measured by qPCR and western blotting.

**Results:**

ATP, MMP, and mPTPs were markedly decreased in COPD rats, while cell apoptosis, caspase-3, and Cyto C were increased (*P*<0.01). All aforementioned parameters were improved in treatment groups (*P*<0.05). ATP, MMP, and mPTPs were significantly higher in the Bufei Jianpi group than in the aminophylline group, while cell apoptosis, caspase-3, and Cyto C were lower (*P*<0.05).

**Conclusions:**

Bufei Jianpi granules can inhibit mitochondrial dysfunction and cell apoptosis in peripheral muscles, which might be the mechanism involved in improving skeletal muscle function in COPD patients.

## 1. Introduction

Chronic obstructive pulmonary disease (COPD) is a major public health problem; this chronic disease is characterized by persistent airflow limitation and is the third leading cause of death in China [1]. As one of the most important systemic complications of COPD, skeletal muscle dysfunction (SMD) is considered a key factor in accelerating the deterioration of the disease; notably, it has a considerable impact on disability and mortality of patients [2, 3]. Indeed, a previous study showed that approximately one-third of COPD patients exhibited quadriceps atrophy in the initial phase of the disease, which led to poorer prognosis [4]. Recently, skeletal muscle cell apoptosis has been recognized as an important mechanism associated with SMD; other important mechanisms include malnutrition, systemic inflammation, and muscular protein synthesis differentiation/regeneration damage. Despite the discoveries of these associations, the specific underlying mechanism remains unclear [5–7].

Apoptosis is defined as programmed cell death. Mitochondria, the primary energy source of cells, play an important role in cellular apoptotic processes [8] and are considered to be central actors in the intrinsic apoptotic pathway. In particular, the aggregation of mitochondria with high Ca^2+^ levels, especially in the context of oxidative stress and low adenine nucleotide, will result in the opening of mitochondrial permeability transition pores (mPTPs) [9, 10]. When mPTPs remain open, mitochondrial swelling occurs, along with reduction of mitochondrial membrane potential (MMP) and discontinuation of ATP synthase activity [11, 12]. Importantly, mPTP opening can result in the release of proteins (normally localized within the mitochondria) into the cytosol; this includes Cytochrome C (Cyto C) and apoptosis protease-activating factor 1 (Apaf-1), which activates caspases that participate in apoptosis [13].

Cyto C, a soluble protein in the inner mitochondrial membrane, is activated by a cascade of events that causes its release into the cytoplasm; subsequently, it triggers cellular apoptosis [14, 15]. Furthermore, caspases play important roles in apoptosis [16]. Caspase-3 is known as an executioner protein and is elevated in apoptotic cells [17]. When initiator caspases are activated by extrinsic or intrinsic apoptosis pathways, procaspase-3 becomes active, eventually leading to cell apoptosis [18].

SMD belongs to the category of atrophy (Weizheng disease) in traditional Chinese medicine (TCM) theory, and the pattern of lung-spleen qi deficiency comprises the primary pathogenesis of skeletal muscle dysfunction in the stable phase of COPD [19, 20]. In TCM theory, qi constitutes the air breathed in and out of the body during respiration, as well as refined nutritious substances flowing in the body (these are known as vital energy). The spleen provides the material basis of the acquired constitution and is the source of both qi and blood; importantly, blood can transport and transform energy (qi) from food and drinks and can strengthen systemic qi, especially that of the lung. In addition, the spleen dominates the muscles and limbs, as well as their functions. When spleen qi deficiency occurs, systemic muscles and limbs become undernourished; thus, their movements and functions are reduced. Therefore, it is important to reinforce the vital qi of spleen and lung for COPD patients, in order to improve their exercise capacity and endurance. Bufei Jianpi granules, a specific prescription for the treatment of lung-spleen qi deficiency syndrome in COPD patients, have been proven clinically effective for improving lung function and quality of life, as well as for reducing frequency of acute exacerbations and prolonging six-minute walk distance; these granules have shown beneficial long-term effects after 1-year follow-up in COPD patients [21]. In a prior study, we found that Bufei Jianpi granules can substantially alleviate the inflammatory response, increase diaphragm muscle function in COPD rats [22], and improve the mitochondrial function and morphometry of peripheral skeletal muscles, such as the quadriceps, intercostal, biceps, and soleus muscles [23].

In this study, we aimed to explore the effects of Bufei Jianpi granules on SMD in rats with COPD that had been induced with repeated cigarette smoke inhalation and bacterial infection by observing the apoptosis of skeletal muscular cells and relevant indicators of mitochondrial function and apoptosis-related mRNAs and proteins.

## 2. Materials and Methods

### 2.1. Animals

Twenty-four male and 24 female Sprague-Dawley (SD) rats were obtained from the Experimental Animal Center of Henan Province (SCXK (Henan): 2015-0005, Zhengzhou, China), all weighing 200 ± 20 g; before experiments were performed, all rats were acclimated by housing them in individual ventilated cases for 7 days in the animal research facility of the First Affiliated Hospital, Henan University of Traditional Medicine (Zhengzhou, Henan, China). The room temperature was maintained at 25 ± 1°C, the relative humidity was 50 ± 10%, and there were 10 to 15 room volume changes per hour; the ammonia concentration in the room was ≤14 mg/m^3^, and the noise level was ≤60 db. The rats had free access to sterilized food and water. All animal studies were conducted in accordance with the Institute's Guide for the Care and Use of Laboratory Animals and were approved by the Experimental Animal Care and Ethics Committee of the First Affiliated Hospital, Henan University of Traditional Chinese Medicine (YFYDW2016027).

### 2.2. Bacteria

*Klebsiella pneumoniae* (strain: 46114) was purchased from the National Center for Medical Culture Collections (Beijing, China) and prepared as a mixed suspension of 6×10^8^ colony-forming units (CFU) before it was administered to rats.

### 2.3. Cigarettes

HongqiQu® Filter cigarettes, containing 10 mg tar, 1.0 mg nicotine content, and 11 mg CO, were provided by Henan Tobacco Industry Co., Ltd. (Zhengzhou, China).

### 2.4. Medicines

Bufei Jianpi granules (comprising Astragali Radix 15 g, Polygonati Rhizoma 15 g, Codonopsis Radix 15 g, Atractylodis Macrocephalae Rhizoma 12 g, Poria 12 g, Fritillariae Thunbergii Bulbus 9 g, Pheretima 12 g, Magnoliae Officinalis Cortex 9 g, Citri Reticulatae Pericarpium 9 g, Asteris Tatarici Radix 9 g, Ardisiae Japonicae Herba 15 g, and Epimedii Herba 6 g) [24] were prepared and provided by the Laboratory of Pharmacology, Henan University of Chinese Medicine (Zhengzhou, China). All herbs were identified by a professional senior pharmacist and prepared in fluid extract in accordance with the standard operating procedure. Aminophylline tablets (0.1 g/tablet; Xinhua, Shandong, China) were crushed and prepared into 1 mg·mL^−1^ solution before they were administered to rats.

### 2.5. Model Preparation

Forty-eight rats were randomized into control, model (COPD), Bufei Jianpi, and aminophylline groups. Six male and 6 female rats were in each group. The COPD rat model was generated by using repeated cigarette smoke inhalation and bacterial infection, while control animals received ventilation with filtered air. Rats were exposed to cigarette-smoke (concentration: 3000 ± 500 ppm) generated from a smoke generator (Buxco, Wilmington, NC, USA) for 30 min, twice per day, at 3-hour intervals.* Klebsiella pneumonia* solution (0.1 mL) was slowly dropped into both nostrils of the rats in an alternating fashion, every 5 days for the first 8 weeks [25].

### 2.6. Administrations and Quadriceps Sampling

From week 9 through week 20, rats underwent intragastric administration of normal saline (2 mL per rat, twice per day) in the control and model groups, Bufei Jianpi granules (4.84 g·kg^−1^·d^−1^, twice per day) in the Bufei Jianpi group, and aminophylline (2.3 mg·kg^−1^·d^−1^, twice per day) in the aminophylline group. The equivalent dosages of Bufei Jianpi granules and aminophylline were calculated by the formula: D_rat_ = D_human_ × (K_rat_/K_human_) × (W_rat_/W_human_)^2/3^, D: dose; K: body shape index, where K = A/W^2/3^ (A: surface area in m^2^, W: weight in kg); W: body weight. All rats were subjected to necropsy after intraperitoneal injection of pentobarbital 100 mg/kg body weight at the end of week 20, and the quadriceps muscle was harvested from each rat. The successful generation of a COPD rat model was evaluated according to symptoms, lung function, and pulmonary pathology [26].

### 2.7. TUNEL Analysis

After formalin lavage and fixation for 72 h, quadriceps tissues were embedded with paraffin and cut into 4-*µ*m slices; they were stained with an In Situ Cell Death Detection Kit (Roche Diagnostics, Mannheim, Germany), in accordance with the manufacturer's instructions. Apoptotic nuclei were visualized using the peroxidase-DAB reaction and then counterstained with hematoxylin. The apoptotic rate was calculated as percentage of TUNEL-positive cells among all cells in the visual field.

### 2.8. Isolation of Mitochondria from Skeletal Muscle

Mitochondria were extracted with the Tissue Mitochondria Isolation Kit (Beyotime, Shanghai, China), in accordance with the manufacturer's instructions. Protein concentration was determined by the BCA Protein Assay kit (Solarbio, Beijing, China).

### 2.9. Measurement of Mitochondrial Membrane Potential

MMP was measured by a fluorescent, lipophilic, cationic probe from the JC-1 kit (Beyotime, Shanghai, China). Briefly, 0.1 mL of depurated mitochondrial suspension, containing 10-100 *μ*g protein, was added into 0.9 mL of JC-1 staining solution (5 *μ*g/mL), and the mixture was incubated at room temperature for 30 min. MMP was determined by measuring the relative amounts of mitochondrial JC-1 monomers or aggregates. The fluorescence intensity of the reaction medium was determined using Varioskan Flash (Thermo Fisher Scientific, Waltham, MA, USA), with excitation wavelength (*λ*_ex_) 485 nm and emission wavelength (*λ*_em_) 590 nm.

### 2.10. Opening of mPTPs

Opened mPTPs of the quadriceps muscle were measured by fluorexon with the mPTP assay kit (Genmed Scientifics Inc., Wilmington, DE, USA), in accordance with the manufacturer's instructions. Briefly, 0.1 mL of depurated mitochondrial suspension, containing 200 *μ*g protein, was added into Reagent A; this mixture was incubated at 37°C for 15 min and centrifuged at 16000 × g for 5 min. The supernatant was discarded and the precipitate was resuspended with Reagent C; the fluorescence intensity of the reaction medium was determined using Varioskan Flash (Thermo Fisher Scientific), with excitation wavelength (*λ*_ex_) 488 nm and emission wavelength (*λ*_em_) 505 nm.

### 2.11. ATP Analysis in Skeletal Muscle

ATP levels were measured using a firefly luciferase based ATP assay kit (Beyotime, Shanghai, China), in accordance with the manufacturer's instructions. Briefly, lysed tissue suspension was centrifuged at 12,000 × g for 5 min. In a 96-well plate, 20 *μ*L of supernatant was mixed with 100 *μ*L ATP detection working dilution. Luminance (in relative light units, RLU) was measured by a monochromator microplate reader (Safire II, Tecan, Switzerland). Protein concentration was determined by the BCA Protein Assay kit (Solarbio). Total ATP levels were expressed as nmol/mg protein.

### 2.12. mRNA Semi-Quantitative Analysis

The expression levels of caspase-3 and Cyto C mRNA in quadriceps tissues were analyzed by semi-quantitative polymerase chain reaction (qPCR). Total RNA was extracted by using a total RNA isolation kit (Solarbio), in accordance with the manufacturer's instructions; the RNA quantity and integrity were verified on a NanoDrop 2000 nano-spectrophotometer (Thermo Fisher Scientific). Reverse transcription (RT) was performed by using Super® III First-Strand Synthesis Super Mix for qRT-PCR Kit (Life Technologies, Grand Island, NY, USA), and real-time PCR reactions were performed by using a Platinum SYBR® Green® Super Mix-UDG Kit (Life Technologies) on an ABI 7300 instrument (Applied Biosystems, Foster City, CA, USA), in accordance with the manufacturer's instructions. Cycling conditions involved an enzyme activation step at 95°C for 2 min, followed by 40 cycles of 95°C for 15 s and 60°C for 30 s. Melting curves ranging from 60°C to 95°C were also included in each run at the end of PCR to evaluate specific amplification of the target genes. The primers for caspase-3 and Cyto C were designed and synthesized by Generay Biotech Co., Ltd. (Shanghai, China); primer sequences are shown in Table 1.

### 2.13. Western Blot Analysis

One hundred milligrams of quadriceps tissues was homogenized in cold RIPA buffer (50 mM Tris-HCl at pH 7.4, 150 mM NaCl, 1% NP-40, 0.25% Na-deoxycholate, and 1 mM phenylmethylsulfonyl fluoride) with freshly added complete protease inhibitor cocktail. All operations were conducted on ice. The homogenates were centrifuged at 4°C at 12,000 × g for 30 min, and total protein concentrations in the supernatant were determined using the Bradford method. Then, 2% sodium dodecyl sulfate (SDS) and 5% 2-mercaptoethanol were added before protein denaturation at 95°C for 5 min. Fifty micrograms of total protein was separated by 10% SDS-polyacrylamide gel electrophoresis and electrotransferred to polyvinylidene difluoride membranes (Millipore, Bedford, MA, USA). The membranes were blocked with 5% bovine serum albumin in Tris-buffered saline containing 20 mmol/L Tris-buffered saline (pH 7.4), 500 mmol/L NaCl, and 0.1% Tween 20 and then incubated with primary antibodies against caspase-3 or Cyto C (Santa Cruz, Dallas, TX, USA), in accordance with the manufacturer's instructions, followed by horseradish peroxidase-conjugated secondary antibodies (Santa Cruz). Finally, signals were detected by using the Super Enhanced Chemiluminescence Plus reagent (Solarbio) and scanned and quantified by a ChemiDoc MP imaging system (BioRad, Hercules, CA, USA).

### 2.14. Statistical Analysis

Data were analyzed by using SPSS Statistics 23.0 software (IBM, Armonk, NY, USA) and are presented as mean ± standard deviation (SD). One-way analysis of variance was performed to detect statistical differences among the groups.* P*<0.05 was considered significant.

## 3. Results

### 3.1. Mortality

Two rats died in the COPD group due to pulmonary infection at the end of week 8, and one died in the Bufei Jianpi group due to pulmonary abscess during week 7.

### 3.2. Cell Apoptosis in the Quadriceps Muscle

**Figure 1:**
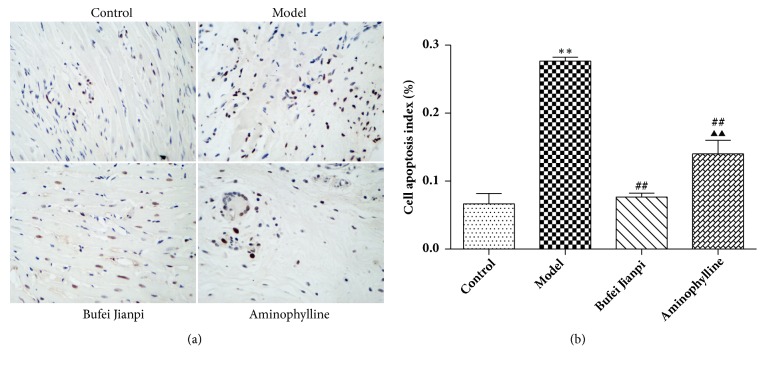
Representative images of cell apoptosis (a) and cell apoptosis rate (b) in quadriceps muscles in rats with chronic obstructive pulmonary disease. Magnification at 400×. N = 10. Values are shown as mean ± standard deviation. ^*∗*^*P*<0.05, ^*∗∗*^*P*<0.01, versus control group; ^#^*P*<0.05, ^##^*P*<0.01, versus model group; ^▲^*P*<0.05, ^▲▲^*P*<0.01, versus Bufei Jianpi group.

As shown in Figure 1, the cell apoptosis rate of the quadriceps muscle was significantly higher in the model group than in the control group (*P*<0.01). Compared with COPD rats, the cell apoptosis rate was significantly lower in the Bufei Jianpi and aminophylline groups (*P*<0.01); it was markedly reduced in the Bufei Jianpi group, compared with the aminophylline group (*P*<0.01).

### 3.3. Mitochondrial Function in the Quadriceps Muscle

**Figure 2:**
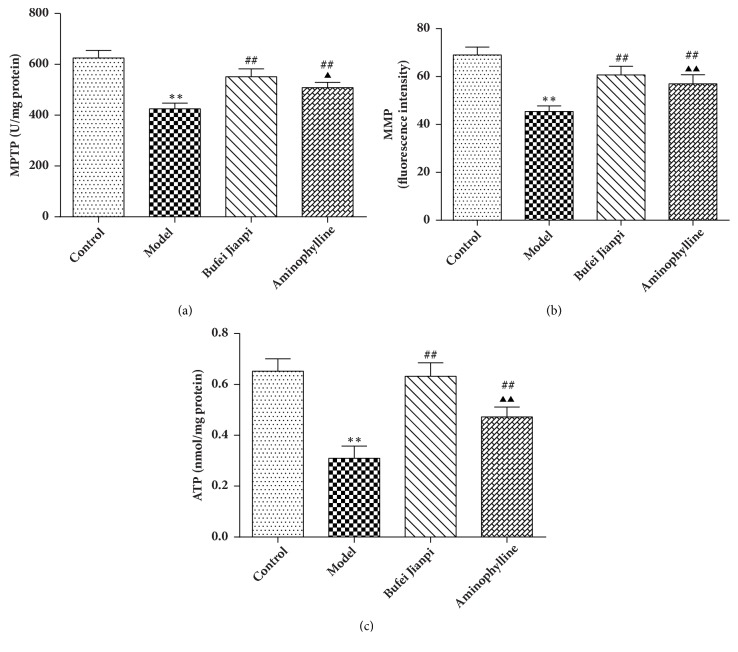
Changes in mPTPs (a), MMP (b), and ATP (c) levels in quadriceps muscle in rats with chronic obstructive pulmonary disease. N = 10. Values are shown as mean ± standard deviation. ^*∗*^*P*<0.05, ^*∗∗*^*P*<0.01, versus control group; ^#^*P*<0.05, ^##^*P*<0.01, versus model group; ^▲^*P*<0.05, ^▲▲^*P*<0.01, versus Bufei Jianpi group.

As shown in Figure 2, mPTPs (Figure 2(a)), MMP (Figure 2(b)), and ATP levels (Figure 2(c)) in the quadriceps muscle were significantly reduced in COPD rats compared with the levels in the control group (*P*<0.01). Compared with the levels in the model group, mPTPs, MMP, and ATP levels increased significantly in the Bufei Jianpi and aminophylline groups (*P*<0.01). All abovementioned indicators of mitochondrial function were markedly elevated in the Bufei Jianpi group, compared with the aminophylline group (*P*<0.05).

### 3.4. mRNA Expression Levels of Caspase-3 and Cyto C in the Quadriceps Muscle

**Figure 3:**
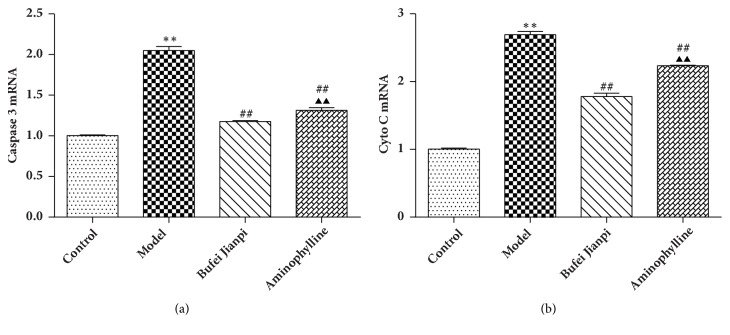
mRNA expression levels of caspase-3 (a) and Cyto C (b) in quadriceps muscles in rats with chronic obstructive pulmonary disease. Values are shown as mean ± standard deviation. ^*∗*^*P*<0.05, ^*∗∗*^*P*<0.01, versus control group; ^#^*P*<0.05, ^##^*P*<0.01, versus model group; ^▲^*P*<0.05, ^▲▲^*P*<0.01, versus Bufei Jianpi group.

As shown in Figure 3, caspase-3 and Cyto C mRNA expression significantly decreased in COPD rats, compared with in controls (*P*<0.01). Compared with the model group, caspase-3 and Cyto C mRNA levels increased significantly in the Bufei Jianpi and aminophylline groups (*P*<0.01). Caspase-3 and Cyto C mRNAs were markedly higher in the Bufei Jianpi group, compared with in the aminophylline group (*P*<0.01).

### 3.5. Protein Expression Levels of Caspase-3 and Cyto C in the Quadriceps Muscle

**Figure 4:**
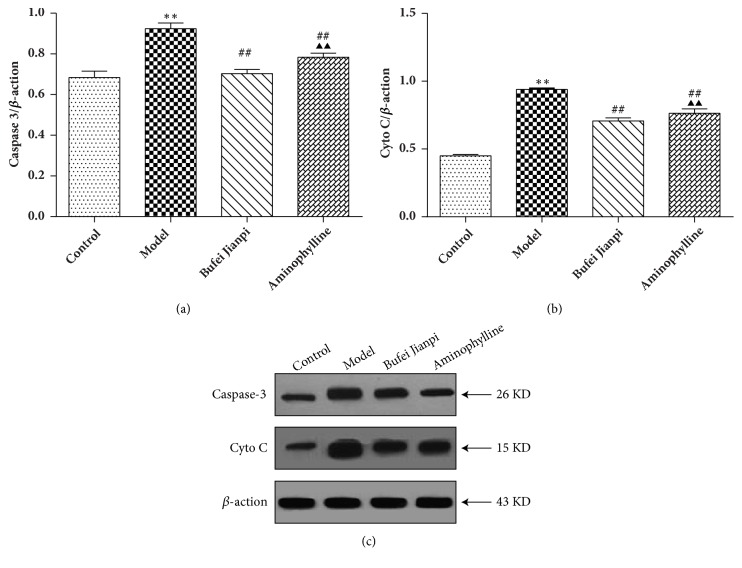
Protein expressions of caspase-3 (a) and Cyto C (b) in quadriceps muscles in rats with chronic obstructive pulmonary disease. Values are shown as mean ± standard deviation. ^*∗*^*P*<0.05, ^*∗∗*^*P*<0.01, versus control group; ^#^*P*<0.05, ^##^*P*<0.01, versus model group; ^▲^*P*<0.05, ^▲▲^*P*<0.01, versus Bufei Jianpi group.

Caspase-3 and Cyto C protein expression levels decreased significantly in COPD rats, compared with those in controls (*P*<0.01). Compared with the model group, caspase-3 and Cyto C protein expression levels increased significantly in the Bufei Jianpi and aminophylline groups (*P*<0.01). Caspase-3 and Cyto C protein expression levels were significantly higher in the Bufei Jianpi group than in the aminophylline group (*P*<0.05) (Figure 4).

## 4. Discussion

This study explored the beneficial effects of Bufei Jianpi granules on skeletal muscle cell apoptosis and mitochondrial dysfunction, using a COPD rat model that was induced with repeated cigarette smoke inhalation and bacterial infection. The results showed that Bufei Jianpi granules can improve mitochondrial function and inhibit cell apoptosis in the quadriceps muscles, which might be the mechanism by which these granules improve skeletal muscle function in COPD patients.

In TCM, SMD is classified as a Weizheng Disease, and it might be a complication of many diseases. COPD is considered to be a debilitating disease and is often combined with SMD during early-stage disease [4]. The pattern of lung-spleen qi deficiency is the main pathogenesis involved in stable COPD [19, 20]. Because the spleen governs production of qi and blood, thereby modifying the activities of muscles and limbs of the body, and the lungs govern qi actions, spleen-lung qi deficiency will result in skeletal muscle dysfunction and atrophy, in addition to the classical respiratory symptoms. Therefore, it is important to reinforce the vital qi of the spleen and lungs, in order to strengthen the exercise capacity and respiratory function of COPD patients. Bufei Jianpi granules, a specific prescription for lung-spleen qi deficiency syndrome, have been confirmed to improve lung function, reducing the frequency of acute exacerbations and prolonging six-minute walk distance; moreover, they have demonstrated beneficial long-term effects after 1 year of follow-up in COPD patients [21]. Our previous experimental studies showed that Bufei Jianpi granules can considerably alleviate the inflammatory response and can increase diaphragm muscular tension and endurance in COPD rats [22]; furthermore, these granules improve the mitochondrial population and morphometry of peripheral skeletal muscles, such as the intercostal, quadriceps, biceps, and soleus muscles [23].

SMD is a key factor in accelerating the deterioration of COPD and has substantial impacts on the disability and mortality of the disease. Indeed, quadriceps muscle dysfunction, primarily characterized by reduced muscle force, is observed in approximately one-third of patients with COPD, even at very early stages of the disease [27]; quadriceps weakness and reduced muscle mass are considered reliable predictors of mortality in COPD prognosis [28].

Skeletal muscle cell apoptosis is an important mechanism that leads to SMD. Research has suggested that cell apoptosis increased in skeletal muscle of COPD patients with low body mass index [29]. Another study indicated that, compared with that in controls, the cell apoptosis rate was significantly elevated in the diaphragms of both moderate and severe COPD patients [30]. In this study, our results showed that cell apoptosis was significantly elevated in COPD rats and that Bufei Jianpi granules could significantly reduce cell apoptosis in quadriceps muscle, to a greater extent than that observed with aminophylline.

Mitochondria are the primary cellular energy source and are involved in the production of ATP, as well as in apoptosis. Mitochondrial dysfunction is recognized as a central aspect of skeletal muscle deterioration in COPD patients; this primarily occurs in the vastus lateralis [8] and is characterized by reduced mitochondrial biogenesis and density and impaired mitochondrial respiration and coupling, as well as increased mitochondrial apoptosis and abnormal autophagy [31, 32]. An important consequence of mitochondrial dysfunction is the activation of apoptosis; indeed, when mitochondria accumulate high concentrations of Ca^2+^, especially in combination with oxidative stress and adenine nucleotide depletion, these stimuli result in the opening of mPTPs [9, 10]. When mPTPs remain open, mitochondrial swelling occurs, as well as reduction of MMP and discontinuation of ATP synthase [11, 12]. Previous studies have shown that mPTPs are open in the skeletal and respiratory muscles of patients with mild to moderate COPD [33, 34]. Our results show that mitochondrial function decreased significantly in the quadriceps muscle in COPD rats. Bufei Jianpi granules can significantly improve mPTPs, MMP, and ATP in quadriceps muscle, along with improving corresponding muscular function; therefore Bufei Jianpi shows a greater benefit than aminophylline.

Cyto C released from the mitochondrion is required for caspase-mediated apoptosis [35]. A prior study showed that mitochondria from the vastus lateralis exhibit abnormal mPTP kinetics (i.e., the time for pore opening was shorter, while the rate of opening was greater) and release larger amounts of Cyto C in patients with COPD [34]. Caspase-3 is an executioner protein in apoptosis, which was found during studies of* Caenorhabditis elegans *development [36]. Increased activation of caspase-3 has been speculated to play a proapoptotic role in the bronchiolar epithelium of smokers with COPD [37]. In the present study, our results showed that the mRNA and protein expression levels of Cyto C and caspase-3 were significantly increased in COPD rats, whereas they were decreased in COPD rats treated with Bufei Jianpi granules. This indicates that Bufei Jianpi granules might improve muscular function by inhibiting mitochondria-dependent apoptotic signaling. Overall, Bufei Jianpi granules show greater benefits than aminophylline for treatment of quadriceps muscle weakness in this COPD rat model.

## 5. Conclusions

Bufei Jianpi granules can improve quadriceps muscular function in a COPD model, particularly with regard to lung-spleen qi deficiency syndrome, by improving mitochondrial function and inhibiting mitochondria-dependent apoptotic signaling, which indicates that this TCM therapy may be effective against debilitating diseases and comorbidities.

## Figures and Tables

**Table 1:** Primer sequences use for real-time PCR assessment of caspase-3 and Cyto C mRNA expression.

Gene		Oligonucleotide primers (5′-3′)
Caspase-3	F	TAACCTCAGAGAGACATTCATGGCC
	R	GAATCACACACACAAAACTGCTCCT

Cyto C	F	TGTTCAAAAGTGTGCCCA
	R	CCAAATACTCCATCAGGG

GAPDH	F	TTTGAGGGTGCAGCGAACTT
	R	ACAGCAACAGGGTGGTGGAC

## Data Availability

The data used to support the findings of this study are available from the corresponding author upon request.

## Abbreviations

COPD: Chronic obstructive pulmonary disease
SMD: Skeletal muscle dysfunction
MMP: Mitochondrial membrane potential
mPTPs: Mitochondrial permeability transition pores
TCM: Traditional Chinese medicine
TUNEL: Transferase-mediated deoxyuridine triphosphate-biotin nick end labeling.
